# A green chemistry approach for oxidation of alcohols using novel bioactive cobalt composite immobilized on polysulfone fibrous network nanoparticles as a catalyst

**DOI:** 10.3389/fchem.2022.1015515

**Published:** 2022-12-20

**Authors:** Andrés Alexis Ramírez-Coronel, Salim Oudah Mezan, Indrajit Patra, Ramaswamy Sivaraman, Yassine Riadi, Shukhrat Khakberdiev, Holya A. Lafta, Munther Abosaooda, Abduladheem Turki Jalil, Yasser Fakri Mustafa

**Affiliations:** ^1^ Laboratory of Psychometrics, Comparative Psychology and Ethology (LABPPCE), Health and Behavior Research Group (HBR), Cuenca, Ecuador; ^2^ General Directorate of Education in Al-Muthanna Governorate, Ministry of Education, Baghdad, Iraq; ^3^ An Independent Researcher, NIT Durgapur, Durgapur, West Bengal, India; ^4^ Department of Mathematics, Dwaraka Doss Goverdhan Doss Vaishnav College, University of Madras, Chennai, India; ^5^ Department of Pharmaceutical Chemistry, College of Pharmacy, Prince Sattam bin Abdulaziz University, Al-Kharj, Saudi Arabia; ^6^ Head of the Chemistry Department, Jizzakh Polytechnic Institute, Jizzakh, Uzbekistan; ^7^ Al-Nisour University College, Baghdad, Iraq; ^8^ College of Pharmacy, The Islamic University, Najaf, Iraq; ^9^ Medical Laboratories Techniques Department, Al-Mustaqbal University College, Hilla, Babylon, Iraq; ^10^ Department of Pharmaceutical Chemistry, College of Pharmacy, University of Mosul, Mosul, Iraq

**Keywords:** green chemistry, nanocatalyst, oxidation of alcohols, microwave-assisted conditions, anticancer activity, cobalt composite immobilized on polysulfone

## Abstract

In this study, cobalt composite immobilized on polysulfone fibrous network nanoparticles (CCPSF NPs) were synthesized in a controllable and one-step way under microwave**-**assisted conditions. The structure of CCPSF NPs was characterized by SEM images (for morphology and size distribution), TGA (for thermal stability), BET technique (for the specific surface area), FT-IR spectroscopy (for relation group characterization), and XRD patterns (for crystal size). The oxidation of the primary and secondary alcohols to aldehyde and ketone was investigated using synthesized CCPSF NPs under solvent-free microwave-assisted conditions, and high oxidizing activity was observed. In addition to oxidation properties, the anticancer activity of the synthesized CCPSF NPs in breast cancer was evaluated by the MTT method , and significant results were obtained.

## 1 Introduction

The increase in the world population causes an increase in the consumption of various substances. As we know, the use of traditional methods in the synthesis of compounds leads to environmental pollution. With the progress of science and technology, new and green methods have taken the place of classical methods. Therefore, the use of green methods leads to the reduction of environmental pollution and high productivity. One of the recently developed methods for the synthesis of green materials is the use of nanoparticles (Appa et al., 2019; Singh et al., 2021; Vallinayagam et al., 2021; Venkateswarlu, 2021; Alshahrani et al., 2022; Naidu and Venkateswarlu, 2022).

Nanomaterials with disordered structures such as carbon nanotubes, oxide structures, composites, and metal–organic frameworks (MOFs) have been synthesized and used for various applications (Wu et al., 2013; Athab et al., 2015; Al-Rowaili et al., 2018; Mohanta et al., 2019; Güemes et al., 2020). In the meantime, many applications such as therapeutic activities, gas storage, separation, and catalytic capabilities of MOF compounds have been reported (Chen et al., 2020; Wu et al., 2020). The review of past literature shows that MOF compounds have biological activities such as antitumor activity, antioxidant, DNA cleavage, antimicrobial, and biofilm inhibition activities (Rojas et al., 2014; Gecgel et al., 2022).

The significant porosity, high specific surface area, and small and uniform particle size can be mentioned among the factors that have affected the importance and applications of these compounds (Ding et al., 2019). Co-precipitation methods (Rani et al., 2020), such as sol–gel (Tarzanagh et al., 2019) and hydrothermal (Zhao et al., 2008) methods, are the methods that have been reported for the synthesis of MOF compounds.

It is very essential to use green and environmentally friendly methods to synthesize these compounds. Since 1986, microwave irradiating technology to speed up the process of chemical reactions has been used. In this method, the reaction is performed in a shorter time with high efficiency, and it is a convenient and effective technique for heating the reaction medium (Lee et al., 2004; Mohammadi et al., 2009; Ghalehbandi et al., 2020).

In the synthesis of MOF compounds, the choice of synthesis method is critical and affects the physical and chemical properties of the products. Reviewing the literature shows that the synthesis of these compounds using microwaves can affect their specific surface and improve their properties (Shu et al., 2020; Ma et al., 2021; Mirhosseini et al., 2021).

As mentioned, one of the applications of MOF compounds is to use them as catalysts. In this field, there have been many reports that these compounds have been used to synthesize organic compounds and polymers (Pascanu et al., 2019; Konnerth et al., 2020).

One of the essential developments in the synthesis of organic compounds is the production of carbonyl compounds by oxidizing alcohols (Ghafuri, 2015; Manesh and Nazari, 2015; Alshammari et al., 2017; Al-Khafaji et al., 2018).

Carbonyl-containing organic compounds by the creation of active intermediates are key chemical compounds for the synthesis of advanced chemicals and effective substances (Reis et al., 2010; Bayat et al., 2015; Sun et al., 2017).

Among the reactions that have recently received attention is the oxidation of alcohols using microwave radiation technology. According to the reactions related to the production of carbonyl compounds with the help of oxidation of alcohols, we conclude that there are still many opportunities to develop methods and achieve simpler, gentler, and environmentally friendly strategies (Hashemi et al., 2004; Ghalehbandi et al., 2020; Alameri and Almashhedy, 2021; Lihumis et al., 2022).

Considering the importance of the oxidation reaction of alcohols and the use of efficient catalysts and green methods, in this research, cobalt composite immobilized on polysulfone fibrous network nanoparticles (CCPSF NPs) were synthesized by a microwave synthesis method and used as catalysts in the oxidation of alcohols under microwave conditions. The advantages of this catalyst are that they oxidize type-1 and type-2 alcohols and diols with higher efficiency and less time and can be reused. Continuing investigations on CCPSF NPs, their anticancer properties were evaluated, and they were also introduced as anticancer agents.

## 2 Experimental Section

### 2.1 Devices and materials

The SEM images were prepared using a Hitachi S-4800 FESEM. A thermal analyzer, STA 409, at a heating rate of 10°C/min was used to record TGA curves. A Nicolet AVATAR 360 FT-IR spectrophotometer was used to obtain the FT-IR spectrum of compounds. Philips XPERT PRO Cu-Kα radiation was used to obtain the XRD pattern of the compound, and finally, a Bruker FT-NMR Ultra Shield spectrometer (250 and 75 MHz) was used to obtain the ^1^H and ^13^C-NMR spectra. For the synthesis of cobalt composite, TAP SONIC (Fanavaran Nano-Meghyas) was used.

An advanced microwave synthesis laboratory station (MicroSYNTH, Milestone Co.) was used for microwave irradiation oxidation of alcohol derivatives.

The solvents and reagents used in this study were prepared by Sigma Aldrich and Merck.

### 2.2 Synthesis of cobalt composite

A mixture of 0.2 mmol of Co(NO_3_)_2_ and 0.6 mmol of pyridine-2,6 dicarboxylic acid in 50 ml of double-distilled water was stirred for 30 min at 70°C. The mixture was placed in an ultrasonicator under a power of 470 W for 20 min at 25°C. Finally, sediment crystals were isolated by centrifugation and nanofiltration, washed several times with acetic acid and water, and dried at room temperature.

### 2.3 Synthesis of cobalt composite immobilized on polysulfone fibrous network nanoparticles

A mixture of 0.05 g of polysulfone powder and 10 mg of cobalt composite was dissolved in 8 ml of acetic acid. A solution was electrospun at a voltage of 25 kV and a spinning distance of 15 cm, and to eject the solutions from the nozzle tip flow, a rate of 5 ml/h was used.

### 2.4 Microwave irradiation oxidation of alcohol derivatives by cobalt composite immobilized on polysulfone fibrous network nanoparticles

A mixture of 10 mmol of alcohol derivatives and 1 mg of CCPSF NPs was stirred at room temperature for 5 min, and then the mixture was irradiated. After completion of the reaction (monitored using thin-layer chromatography), the combinations were cooled, and CCPSF NPs were separated by nanofiltration. To reuse the catalyst, after its separation, it was washed several times with double-distilled water and ethanol and dried under vacuum at 100°C. Finally, for a pure product, crude was passed through a short silica gel column with ethyl acetate:ether (1:7) as solvent.

#### 2.4.1 Aldehyde derivatives

*1-Octanal (6B)*; IR (KBr) = 2,999, 2,841, 1725, and 1,487 cm^−1^; ^1^H NMR (250 MH*z,* CDCl_3_) *δ* = 9.48 (t, 1H), 2.31–238 (m, 2H), 1.42–1.49 (m, 2H), 1.22–1.27 (m, 8H), and 0.84 (t, 3H); ^13^C NMR (75 MH*z,* CDCl_3_) *δ* = 191.86, 42.75, 31.66, 30.24, 29.41, and 23.94.

*4-Methoxybenzaldehyde (12B)*; IR (KBr) = 3,021, 2,948, 2,799, 1721, 1,646, 1,499, 1,320, 1,199, and 841 cm^−1^; ^1^H NMR (250 MH*z,* DMSO) *δ* = 9.91 (s, 1H), 7.65 (d, 2H, J = 8.4 Hz), 7.09 (d, 2H, J = 8.6 Hz), and 3.54 (s, 3H); ^13^C NMR (75 MH*z,* CDCl_3_) *δ* = 191.21, 131.59, 130.54, 114.79, and 55.26.

#### 2.4.2 Ketone derivatives

*2-Methylcyclopentaone (2D)*; IR (KBr) = 3,431, 2,901, 2,838, 1728, 1,427, 1,254, 1,137, and 876 cm^−1^; ^1^H NMR (250 MH*z,* CDCl_3_) *δ* = 2.51 (m, 1H), 2.34 (m, 1H), 2.09 (m, 1H), 1.81 (m, 1H), 1.58 (m, 1H), 1.49 (m, 1H), and 1.19 (m, 1H); ^13^C NMR (63 MH*z,* CDCl_3_) *δ* = 218.13, 44.01, 37.56, 33.45, 21.94, and 15.46.

*Acetophenone (4D)*; IR (KBr) = 3,140, 3,054, 1701, 1,621, 1,354, 1,246, and 778 cm^−1^; ^1^H NMR (250 MH*z,* CDCl_3_) *δ* = 7.39–7.52 (m, 5H) and 2.31(s, 3H); ^13^C NMR (75 MH*z,* CDCl_3_) *δ* = 193.17, 135.82, 131.45, 127.99, 125.09, and 26.76.

#### 2.4.3 Diketone derivatives

*Acetylacetone (1F)*; IR (KBr) = 3,326, 2,458, 1728, and 1,614 cm^−1^; ^1^H NMR (250 MH*z,* DMSO) *δ* = 3.51 (s, 2H), 1.96 (s, 6H), and [15.66(O-H), 5.47 (vinyl H in enol form)]; ^13^C NMR (75 MH*z,* DMSO-d6) *δ* = 200.07, 57.95, 31.42, and [191.35, 101.21, 31.17, 22.86, in enol form].

*2-Aminoanthraquinone (2F)*; IR (KBr) = 3,495, 1,676, 1,433, 1,298, 1,251, 1,149, 1,069, and 961, 777 cm^−1^; ^1^H NMR (250 MH*z,* DMSO) *δ* = 6.51–7.66 (m, 8H) and 2.21 (2H); ^13^C NMR (75 MH*z,* DMSO-d6) *δ* = 189.13, 178.21, 154.66, 135.27, 134.84, 134.18, 131.04, 126.95, 122.43, 118.79, and 110.07.

### 2.5 Anticancer activity

Anticancer activity studies of CCPSF NPs using the MTT method and previously reported on MCF-7 breast cancer cells were done. The densities of 1.2 × 10^4^ (cells/well) MCF-7 breast cancer cells for 24 and 48 h in concentrations of 5, 10, 20, 40, 80, 120, and 200 mg/ml CCPSF NPs were tested (Moghaddam-Manesh and Hosseinzadegan, 2021).

## 3 Results and discussion

### 3.1 Characterization of cobalt composite immobilized on polysulfone fibrous network nanoparticles

**SCHEME 1:**
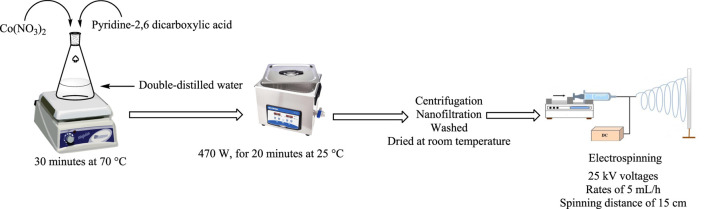
Synthesis of cobalt composite immobilized on polysulfone fibrous network nanoparticles (CCPSF NPs) using ultrasonic and electrospinning methods.

Using ultrasonic and electrospinning methods, according to Scheme 1, cobalt composite immobilized on polysulfone fibrous network nanoparticles (CCPSF NPs) were synthesized.

To identify and confirm the structure of cobalt composite immobilized on polysulfone fibrous network nanoparticles (CCPSF NPs), SEM images for morphology and size distribution, TGA for thermal stability, BET technique for specific surface area, FT-IR spectroscopy for relation group characterization, and XRD patterns for the crystal size were used.

**FIGURE 1:**
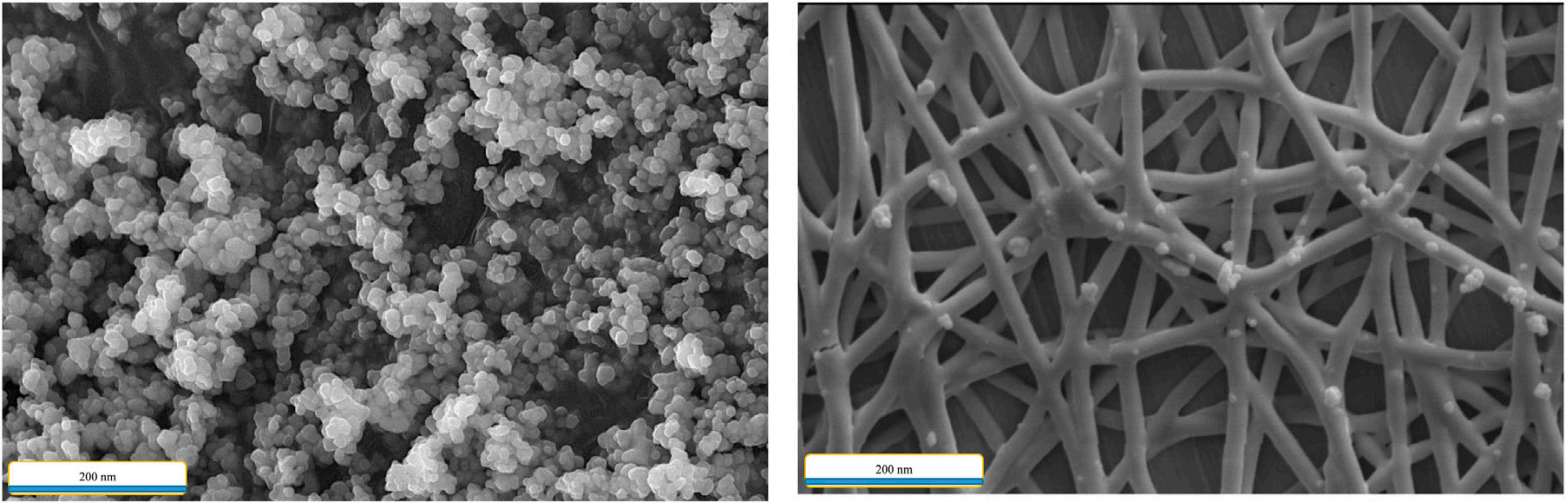
SEM image of (I) cobalt composite and (II) CCPSF NPs.

SEM images of cobalt composite and CCPSF NPs are given in Figure 1.

Figure 1 shows that the size of the nanoparticles is in the nano range and has the same fiber morphology.

**FIGURE 2:**
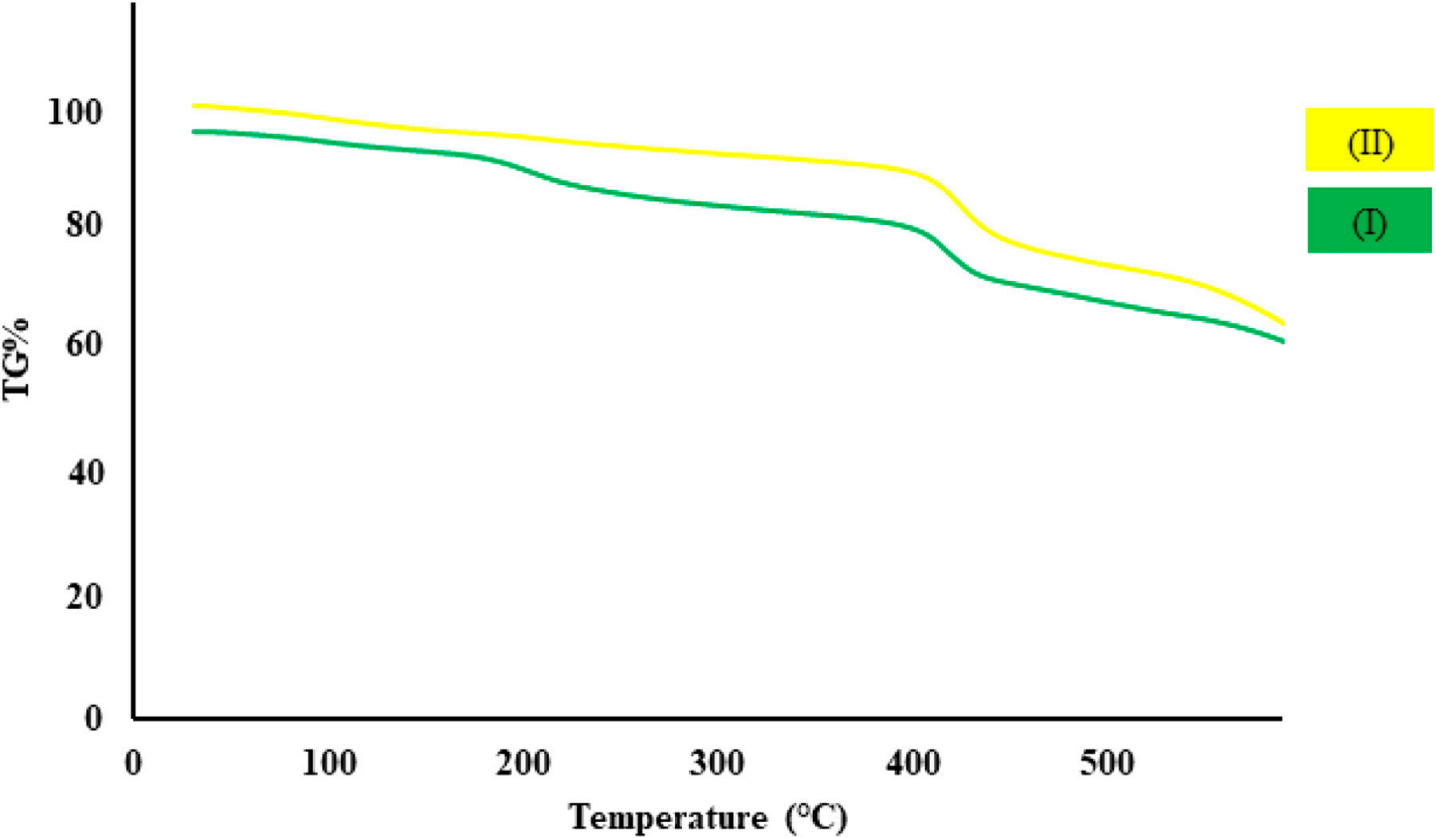
Thermal stability curve of synthesized cobalt composite (I) and CCPSF NPs (II).

As we know, thermal stability is one of the practical factors in designing MOF nanostructures for application in various fields (Ding et al., 2019). The thermal stability curve of synthesized cobalt composite (I) and CCPSF NPs (II) are displayed in Figure 2. The thermal stability of CCPSF NPs was obtained at around 400°C. The high thermal stability of CCPSF NPs shows their high catalytic ability.

**FIGURE 3:**
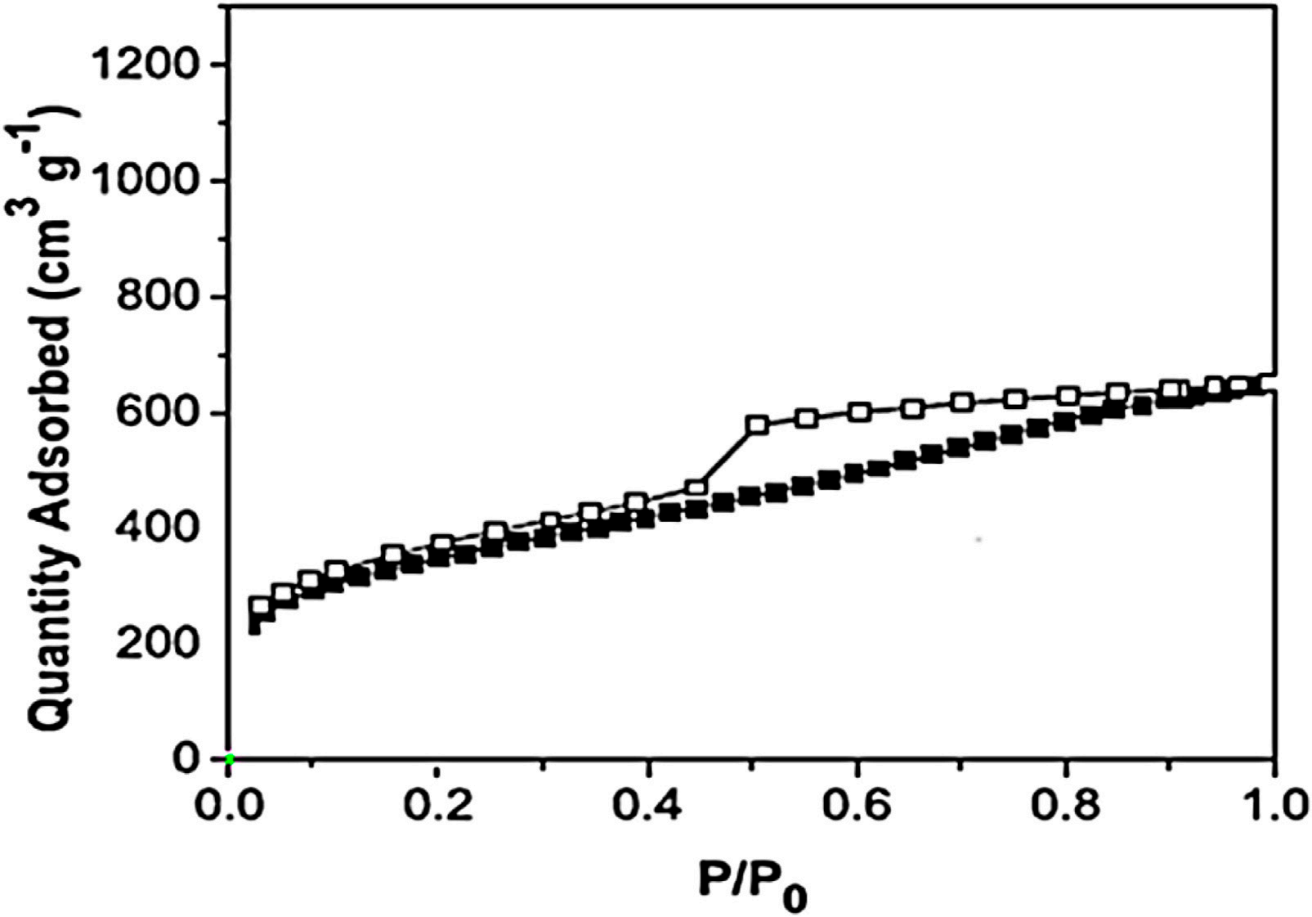
N_2_ adsorption/desorption of CCPSF NPs.

The specific surface area of CCPSF NPs by N_2_ adsorption/desorption isotherms and BET technique 2,450 m^2^/g was obtained (Figure 3). The specific surface area obtained for CCPSF NPs proves that this compound has a high capability in the contact surface with combinations and use as a catalyst. It also seems that CCPSF NPs with an increased specific contact surface can create an increased contact surface with microbial agents and create a high effect.

**FIGURE 4:**
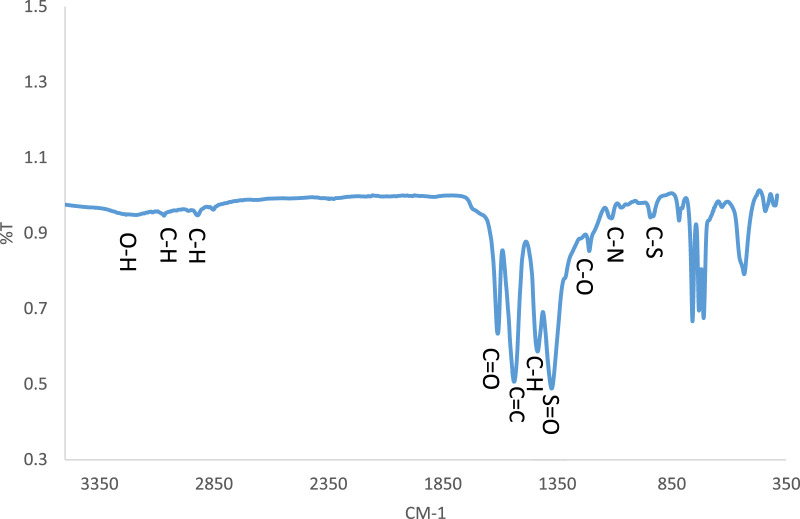
FT-IR spectrum of CCPSF NPs.

Based on the FT-IR spectrum (Figure 4), the peaks of the groups present in the structure of CCPSF NPs were observed. The peak in region 3,300 cm^−1^ was related to hydroxyl (OH) groups. Stretching peaks of C–H groups were shown near 3,067 cm^−1^ and 2,919 cm^−1^. The peak due to carbonyl groups (CO) was near 1,610 cm^−1^. The peak of C=C was at 1,538 cm^−1^. The bending peak of C–H was 1,435 cm^−1^. The S=O groups showed a peak in1370 cm^−1^. C–O, C–N, and C–S groups showed peaks at 1,206, 1,106, and 810 cm^−1^, respectively (Moghaddam-Manesh et al., 2020). The absorption due to Co–O was near 710 and 530 cm^−1^ (Hafeez et al., 2020).

**FIGURE 5:**
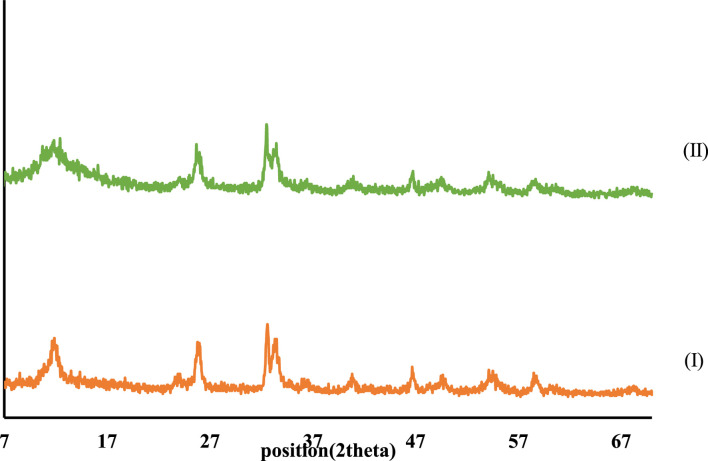
XRD patterns of synthesized cobalt composite (I) and CCPSF NPs (II).

XRD patterns of CCPSF NPs are shown in Figure 5. XRD patterns obtained for CCPSF NPs are similar to XRD patterns reported for cobalt nanoparticles (Raza et al., 2016). The crystal size of CCPSF NPs obtained using the Debby Scherrer equation was about 31 nm.

**FIGURE 6:**
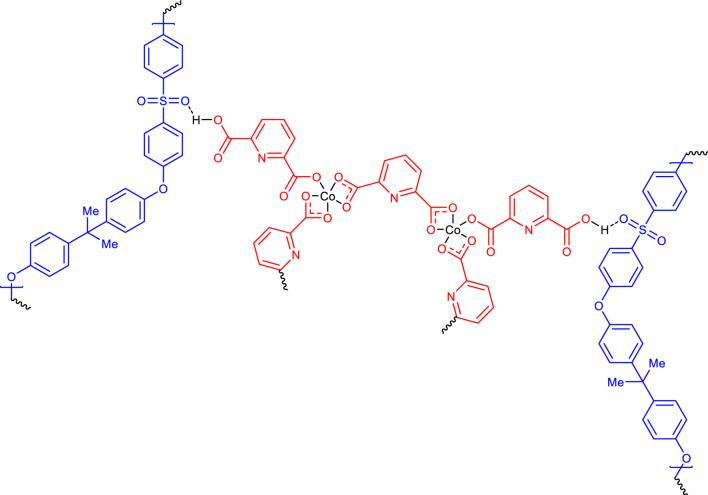
Proposed possible structure for CCPSF NPs.

The structure of Figure 6 was consistent for synthesized CCPSF NPs based on the analyses carried out.

### 3.2 Results of microwave irradiation oxidation of alcohol derivatives by cobalt composite immobilized on polysulfone fibrous network nanoparticles as nanocatalyst

**SCHEME 2:**
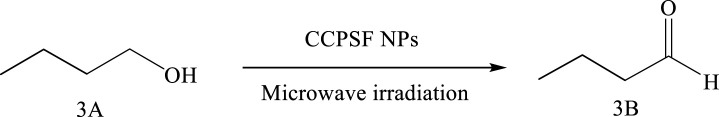
Microwave irradiation oxidation of 1-butanol to 1-butanal using CCPSF NPs as nanocatalysts.

In this research, for the oxidation of alcohol derivatives by microwave irradiation and **CCPSF NPs** as nanocatalysts, first, reaction conditions such as MW power and amount of catalyst for 1-butanol to 1-butanal were optimized (Scheme 2).

The results of the optimization of 1-butanol to 1-butanal as a sample are given in Table 1.

**TABLE 1:** Optimization conditions in the oxidation of 1-octanol to 1-octanal using CCPSF NPs as nanocatalysts under MW irradiation.

Entry	mg catalyst	MW power (W)	Irradiation time (min)	Yield
1	1	—	reflux (12 h)	61
2	1	300	10	72
3	1	400	5	91
**4**	**1**	**500**	**2**	**95**
5	2	500	2	95
6	3	500	5	89
7	4	500	10	85
8	5	500	20	71

That the bold values indicates the selection of optimal conditions.

The optimal conditions: 1 mg of catalyst (CCPSF NPs) and power of microwave irradiation of 500 W for 2 min were used.

**SCHEME 3:**
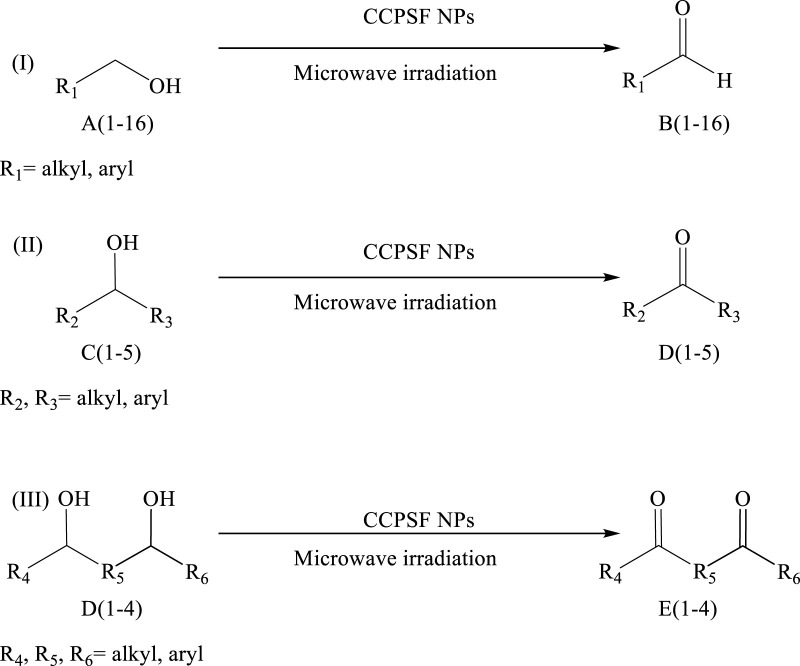
Microwave irradiation oxidation of primary alcohol derivatives (I), secondary alcohol derivatives (II), and diol derivatives (III) using CCPSF NPs as nanocatalysts.

Using CCPSF NPs as nanocatalysts and microwave irradiation, oxidation of primary alcohol derivatives (Scheme 3-I), secondary alcohol derivatives (Scheme 3-II), and diol derivatives (Scheme 3-III) to aldehyde derivatives, ketone derivatives, and diketone derivatives, respectively, was studied.

The rest of the primary alcohol derivatives, secondary alcohol derivatives, and diol derivatives studied in this research were oxidized using optimal conditions according to Tables 2–4.

**TABLE 2:** Green oxidation of primary alcohol derivatives to aldehyde derivatives using CCPSF NPs under MW irradiation.

	Primary alcohol (A)	Aldehyde (B)	Time (min)	Yield (%)	Found M. P. ( °C )	Reported M. P. ( °C ) (Ghalehbandi et al., 2020)
1	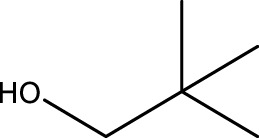	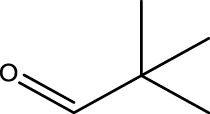	2	91	Liq.	Liq.
2	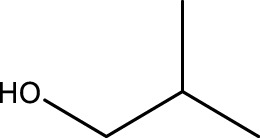	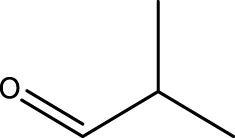	3	92	Liq.	Liq.
3	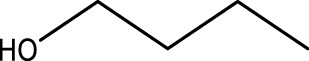	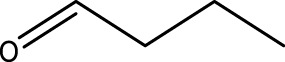	2	90	Liq.	Liq.
4	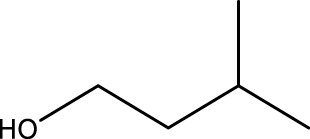	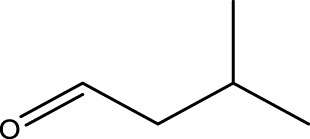	2	91	Liq.	Liq.
5	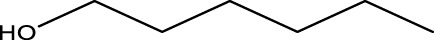	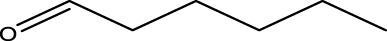	2	91	Liq.	Liq.
6	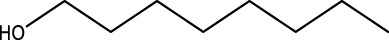	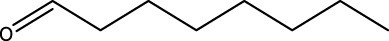	2	95	Liq.	Liq.
7	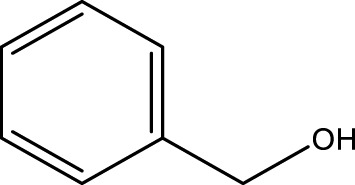	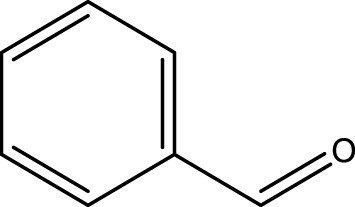	2	100	Liq.	Liq.
8	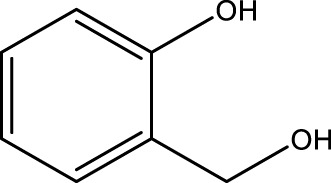	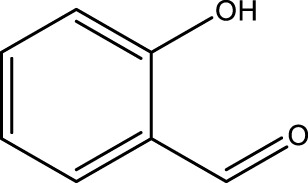	2	100	Liq.	Liq.
9	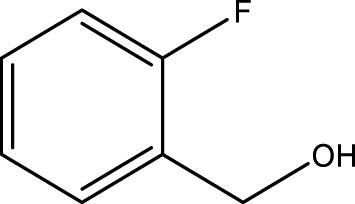	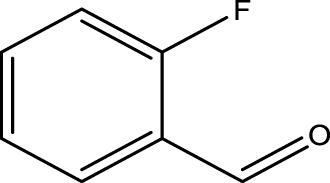	2	93	Liq.	Liq.
10	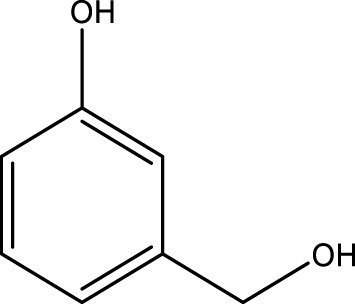	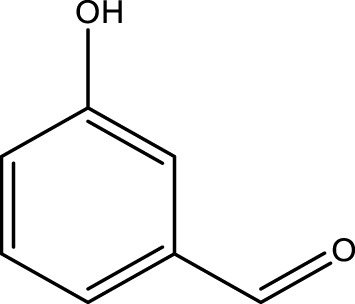	2	98	101–103	104
11	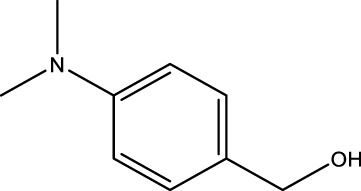	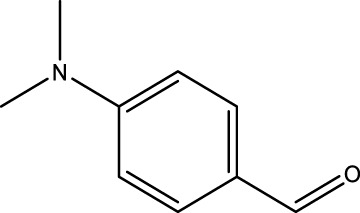	2	97	76–77	77
12	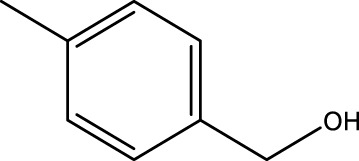	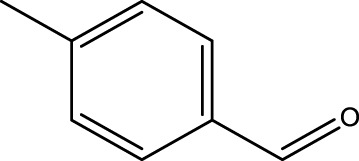	2	100	Liq.	Liq.
13	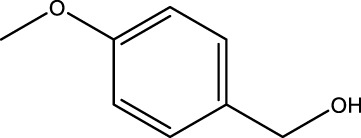	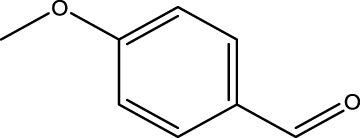	2	100	Liq.	Liq.
14	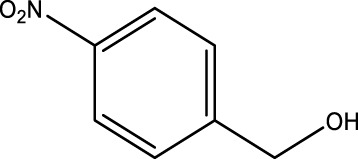	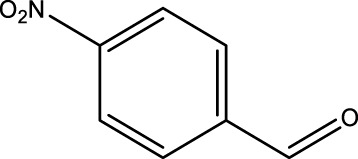	2	96	103–106	104–105
15	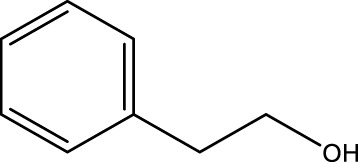	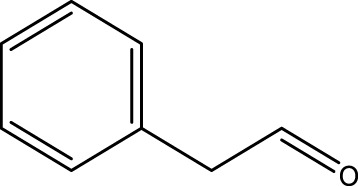	2	95	Liq.	Liq.
16	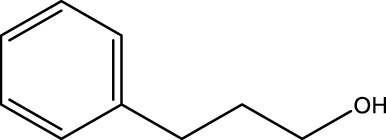	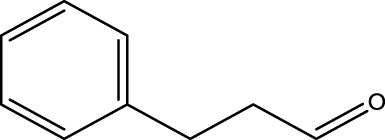	3	92	Liq.	Liq.

Optimal conditions: 1 mg of CCPSF NPs and power of microwave irradiation 500 (W).

**TABLE 3:** Green oxidation of secondary alcohol derivatives to ketone derivatives using CCPSF NPs under MW irradiation.

	Secondary alcohol (C)	Ketone (D)	Time (min)	Yield (%)	Found M. P. ( °C )	Reported M. P. ( °C ) (Ghalehbandi et al., 2020)
1	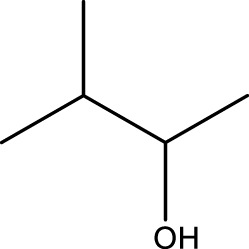	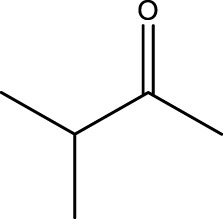	3	91	Liq.	Liq.
2	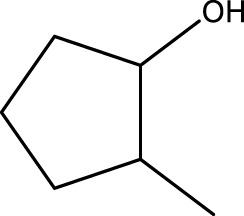	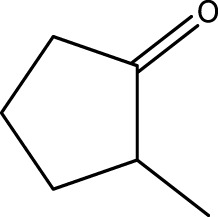	4	92	Liq.	Liq.
3	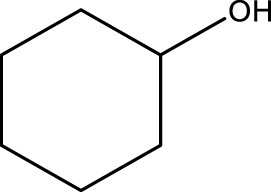	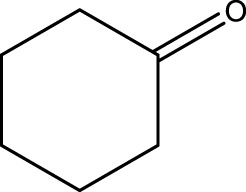	3	93	Liq.	Liq.
4	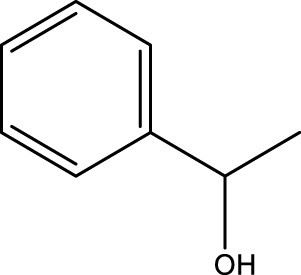	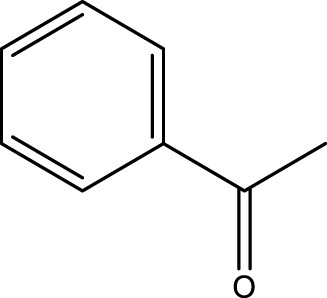	2	100	Liq.	Liq.
5	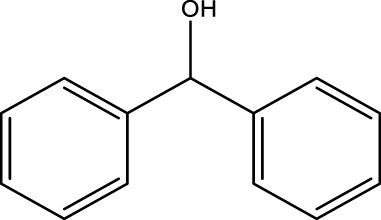	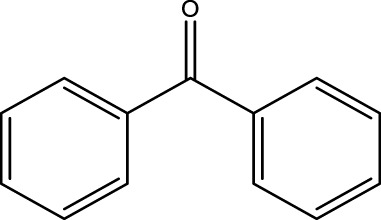	2	97	44–46	45

Optimal conditions: 1-mg of CCPSF NPs and power of microwave irradiation 500 (W).

**TABLE 4:** Green oxidation of diol derivatives to diketone derivatives using CCPSF NPs under MW irradiation.

	Diol (E)	Diketone (F)	Time (min)	Yield (%)	Found M. P. ( °C )	Reported M. P. ( °C ) (Ghalehbandi et al., 2020)
1	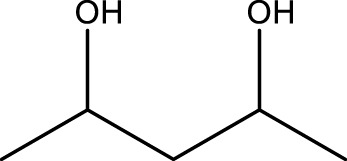	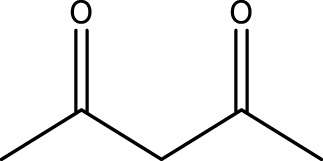	3	93	Liq.	Liq.
2	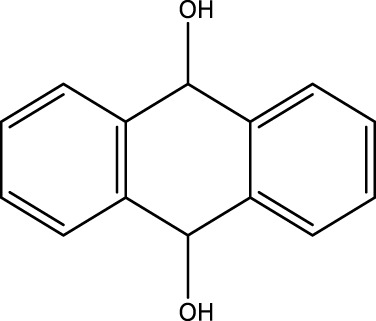	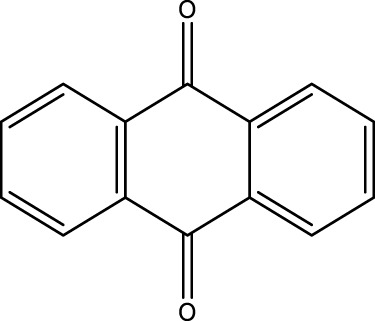	2	100	282	280
3	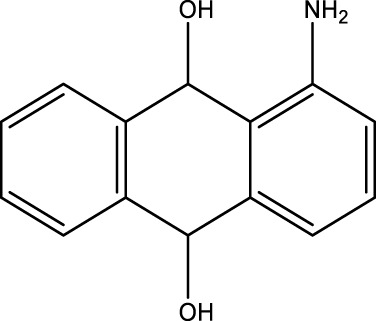	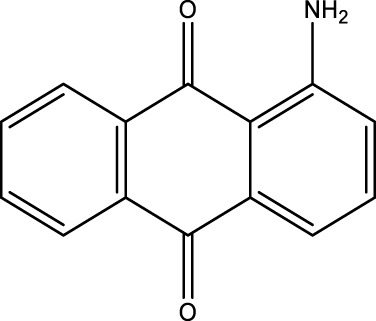	3	95	251–252	250–252
4	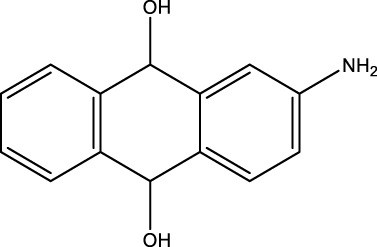	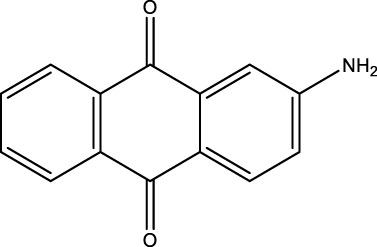	4	93	290–293	290

Optimal conditions: 1 mg of CCPSF NPs and power of microwave irradiation 500 (W).

The results of Tables 2–4 showed that CCPSF NPs have a high ability to oxidize primary alcohol derivatives, secondary alcohol derivatives, and diol derivatives in optimal conditions with high efficiency.

The main advantage of the oxidation reaction using microwave irradiation and CCPSF NPs as nanocatalysts was that the reaction was carried out in solvent-free microwave-assisted conditions, and the reaction conditions were green.

**FIGURE 7:**
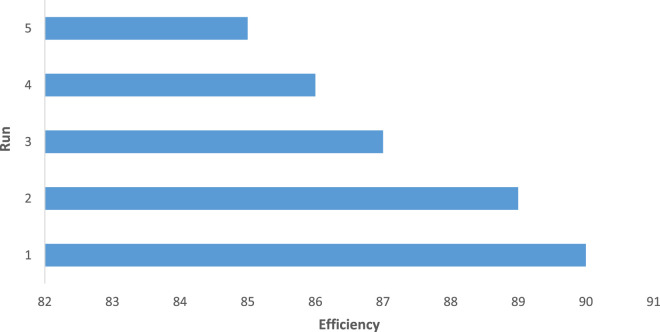
Reusability of CCPSF NPs in the oxidation of benzyl alcohol (row 13 of Table 2)

Another advantage of the CCPSF NPs was their reusability, which was used up to five times (for 1-butanol). The results presented in Figure 7 show that the reaction efficiency did not change much after reuse. For leaching, after the catalyst was reused, a hot filtration test was done, and no enhancement in conversion was noticed in the filtrate.

**SCHEME 4:**
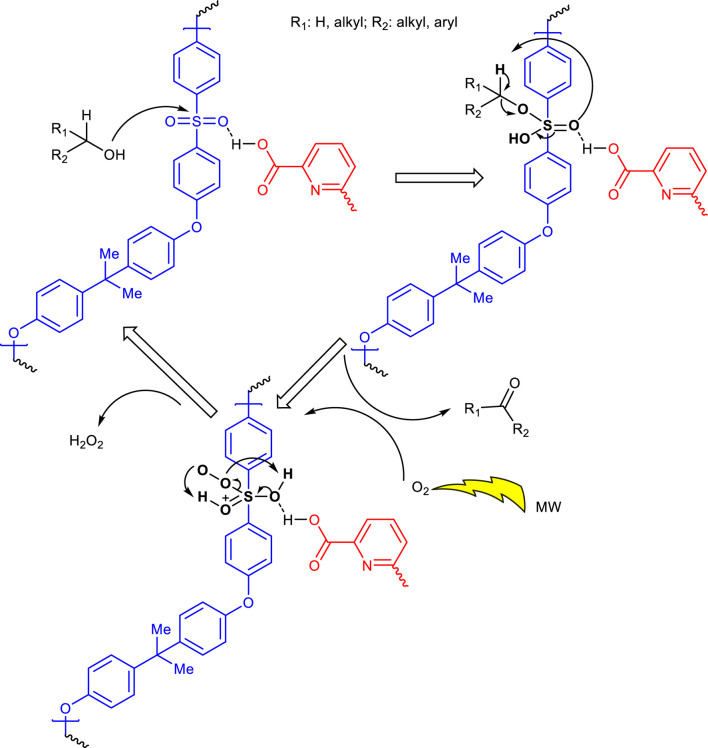
Suggested mechanism for the oxidation of alcohols using CCPSF NPs as the catalyst.

The proposed mechanism for the oxidation of alcohols using CCPSF NPs as catalyst is presented in Scheme 4.

Some of the recently reported methods for the oxidation of alcohols were using Pd@TiC (Bhaumik et al., 2016), silica-supported DABCO tribromide (Moghaddam et al., 2013), platinum (IV) complex (El-Bendary et al., 2022), and Rh/NAC catalysts (Verma et al., 2017) as catalysts. Table 5 compares some of the reported methods for the oxidation of benzyl alcohol in this study.

**TABLE 5:** Different catalysts recently reported for the oxidation of benzyl alcohol.

Entry	Reaction condition	Yield (%)	Time	Ref.
1	Pd@TiC	97	8 (h)	Bhaumik et al. (2016)
2	Silica-supported DABCO tribromide	95	1 (h)	Moghaddam et al. (2013)
3	Rh/NAC catalysts	50	24 (h)	Verma et al. (2017)
4	This study (CCPSF NPs)	100	2 (min)	—

Comparing the results proves that CCPSF NPs can oxidize benzyl alcohol with higher efficiency in less time.

### 3.3 Anticancer activity

**FIGURE 8:**
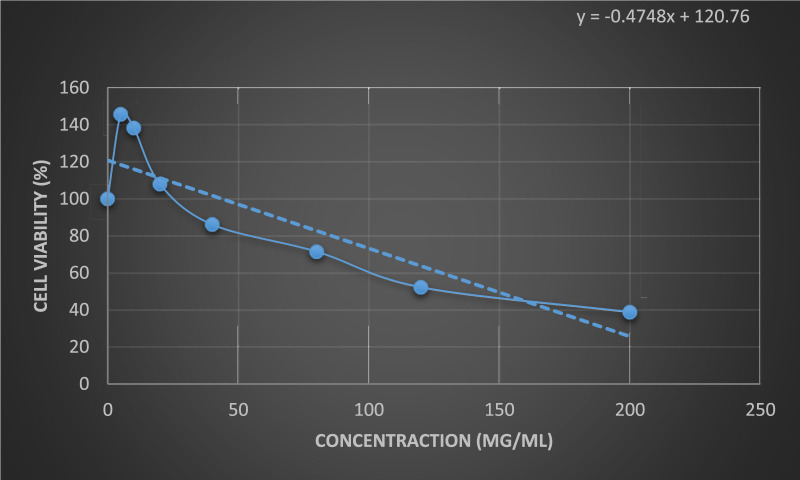
Anticancer activity of CCPSF NPs against MCF-7 breast cancer cells at 24 h. Data represented mean (*n* = 3) ± SD.

**FIGURE 9:**
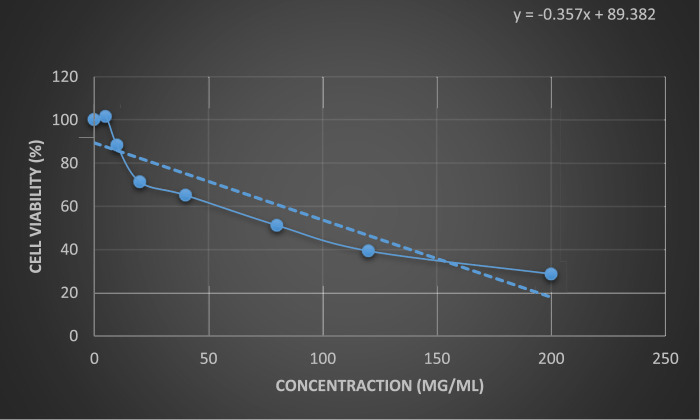
Anticancer activity of CCPSF NPs against MCF-7 breast cancer cells at 48 h. Data represented mean (*n* = 3) ± SD.

**FIGURE 10:**
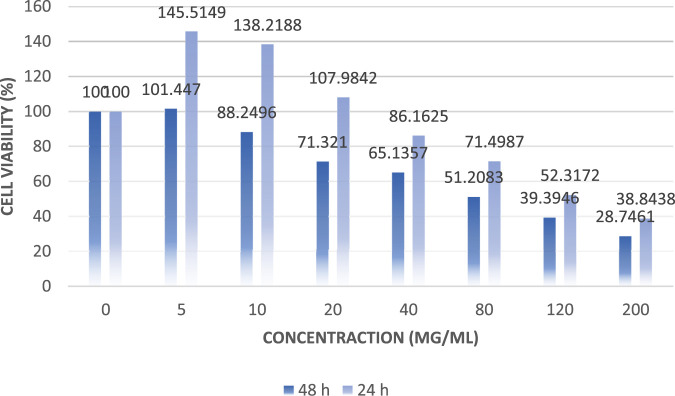
Comparing the results of anticancer activity of CCPSF NPs against MCF-7 breast cancer cells in 24 and 48 h.

The results of the anticancer activity of CCPSF NPs are shown in Figures 8–10.

The IC_50_ values of CCPSF NPs at 24 and 48 h, 149.0312 and 110.3137 mg/ml, respectively, were obtained. The cell proliferation and viability were then controlled at a concentration of 200 mg/ml at 24 and 48 h, and 38.9% and 28.8%, respectively, were observed. The comparison of 24 and 48 h is shown in Figure 10.

Based on the obtained results, it can be concluded that the effect of CCPSF NPs on MCF-7 breast cancer cells depends on concentration and time, and the impact increases with increasing concentration and time.

The anticancer activity of CCPSF NPs can be attributed to the presence of polysulfone (with high biological properties) and cobalt in their structure (Mohamad et al., 2019; Alkış et al., 2021; Chen et al., 2021), as well as its high specific surface area, which was created as a result of the appropriate synthesis method.

## 4 Conclusion

In the present research, using ultrasonic-assisted and electrospinning methods, cobalt composite immobilized on polysulfone fibrous network nanoparticles (CCPSF NPs) were synthesized. After confirming the structure of CCPSF NPs, it was proved that the synthesis method resulted in the synthesis of nanoparticles with a high specific surface area. The high specific surface area of CCPSF NPs made it possible for it to be used as a green, efficient, and reusable nanocatalyst in the oxidation of primary alcohols, secondary alcohols, and diols using microwave irradiation. The obtained results of oxidation of alcohols using CCPSF NPs were higher efficiency and less time required compared to previously reported methods. Among the other capabilities of CCPSF NPs that can be mentioned are their biological properties. In the biological evaluation of nanoparticles, anticancer properties against MCF-7 breast cancer cells were investigated. High effectiveness was observed, which can be attributed to the presence of polysulfone and cobalt in the structure and the high specific surface area of CCPSF NPs.

## Data Availability

The original contributions presented in the study are included in the article/Supplementary Material; further inquiries can be directed to the corresponding author.
