# Supplementary material for: A green chemistry approach for oxidation of alcohols using novel bioactive cobalt composite immobilized on polysulfone fibrous network nanoparticles as a catalyst
Source: Front Chem. 2022 Dec 20;10:1015515. doi: 10.3389/fchem.2022.1015515 (PMC9807923; doi:10.3389/fchem.2022.1015515)
Supplement: Supplementary file 1 [file DataSheet1.docx]

**A green chemistry approach for oxidation of alcohols using novel bioactive cobalt composite immobilized on polysulfone fibrous network as a Catalyst.**

Andrés Alexis Ramírez-Coronel, Salim Oudah Mezan, Indrajit Patra, Ramaswamy Sivaraman, Yassine Riadi, Shukhrat Khakberdiev, Holya A. Lafta, Munther Abosaooda, Abduladheem Turki Jalil, Yasser Fakri Mustafa*

**1-Octanal (6B)**

**^
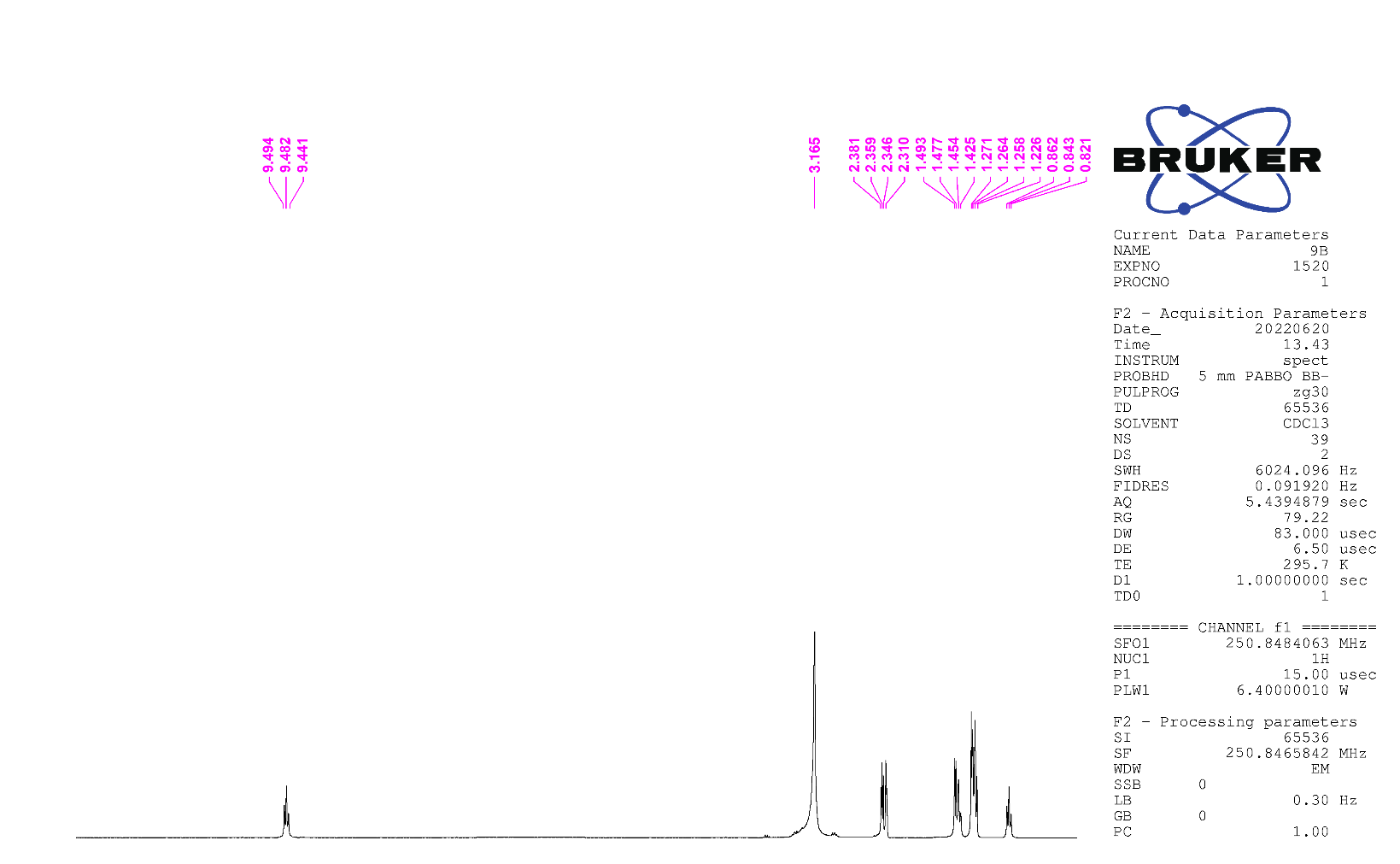
^**


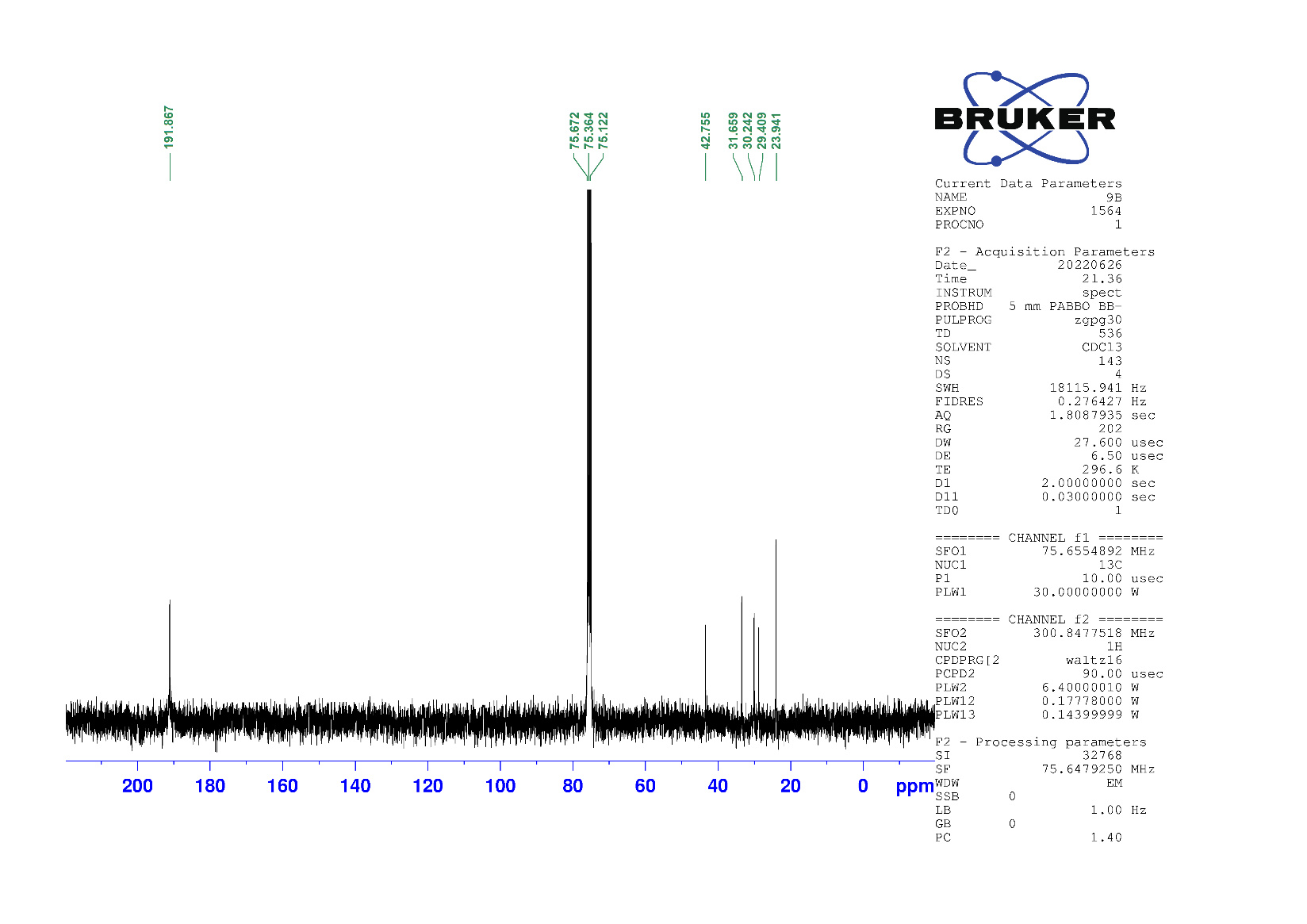


**Acetophenone (4D)**

**
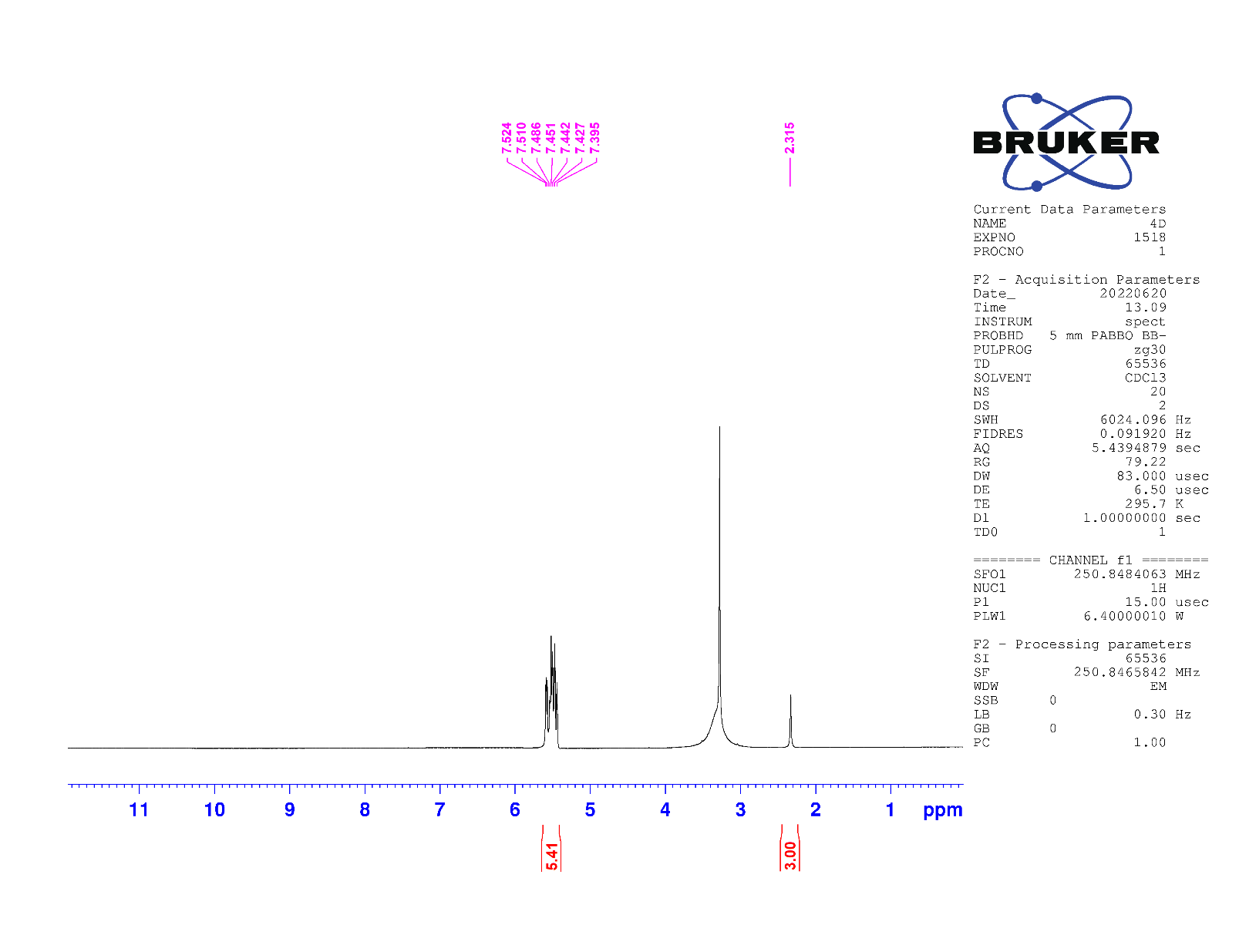
**

**
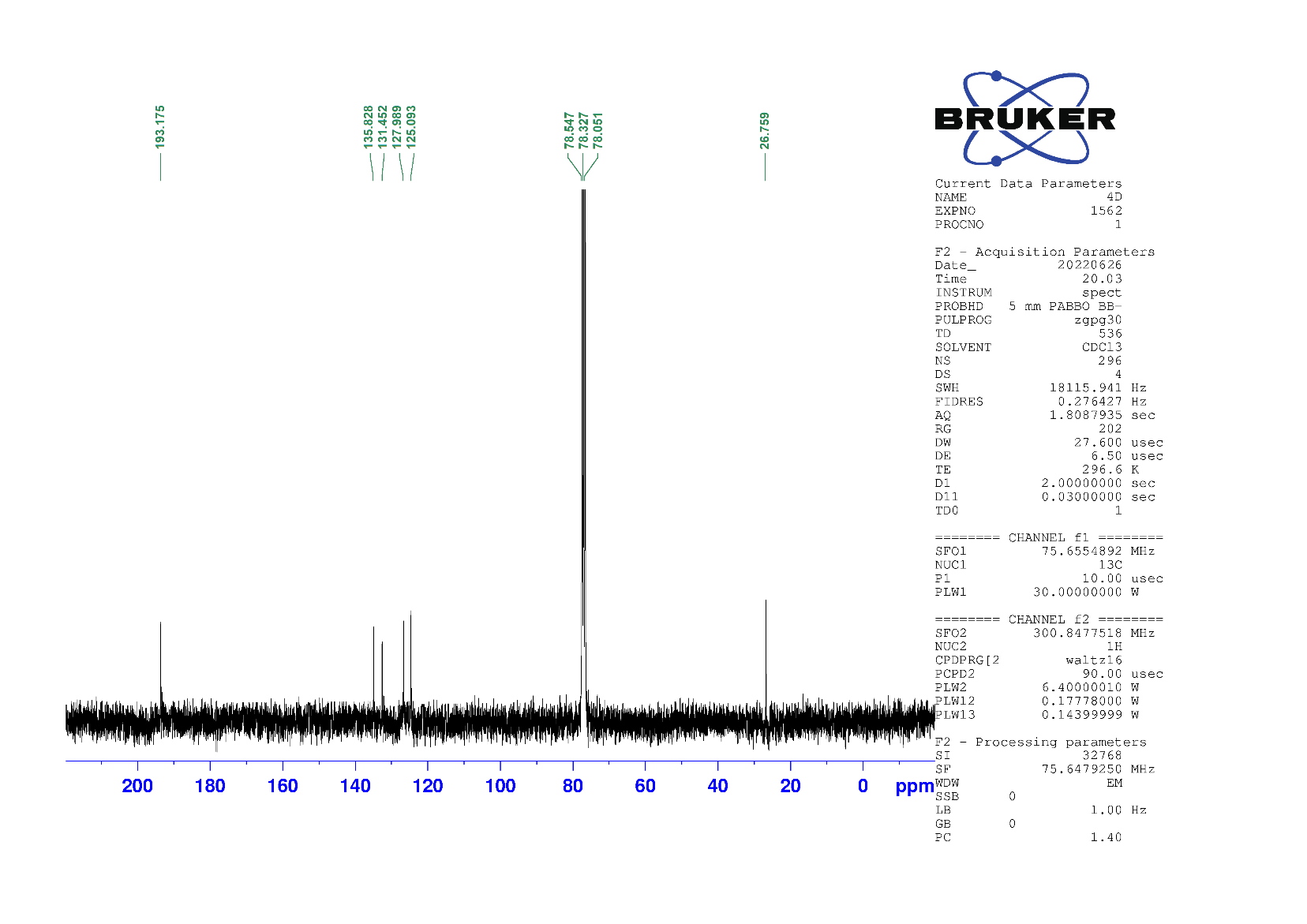
**

**Acetylacetone (1F)**

**
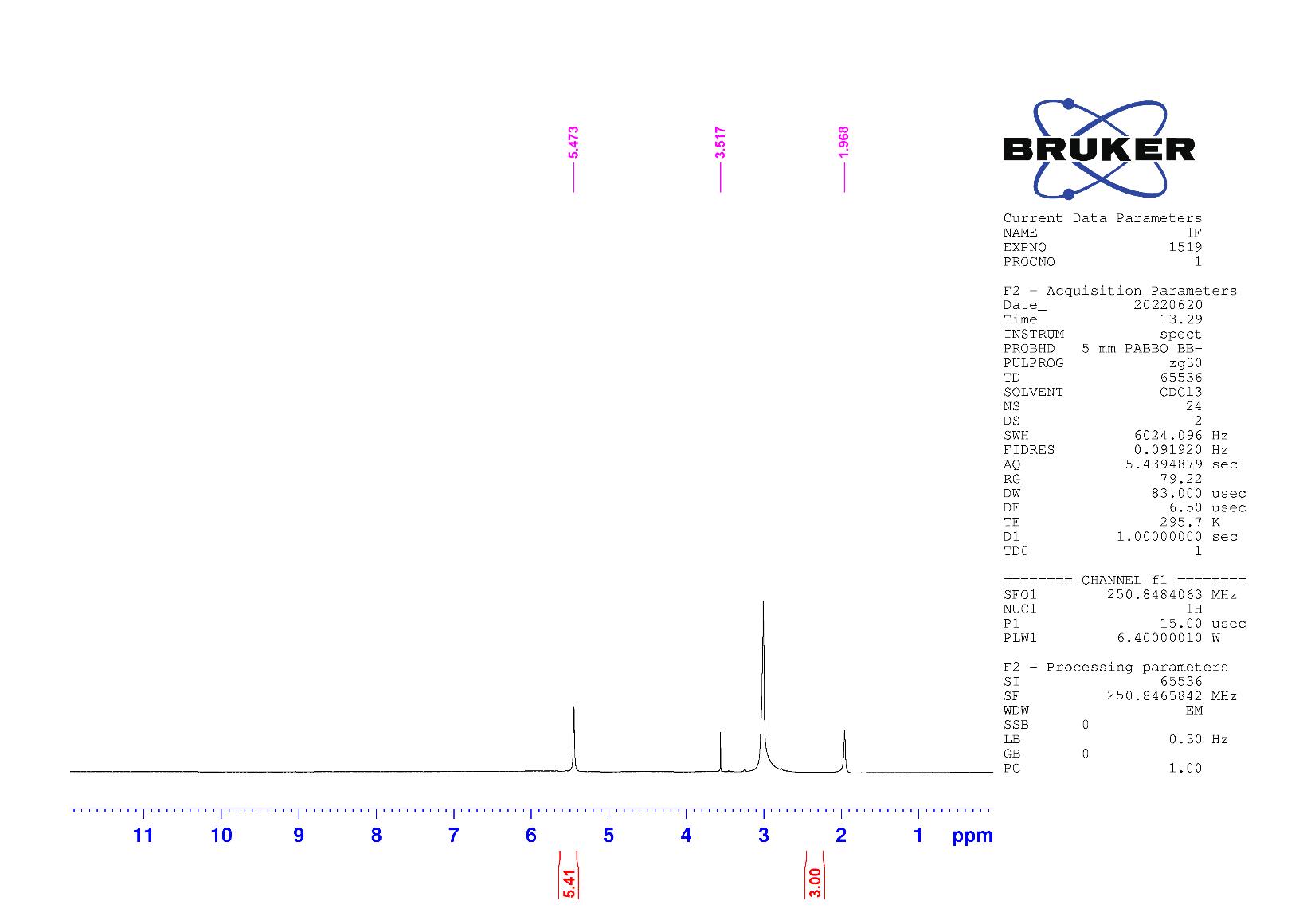
**

**
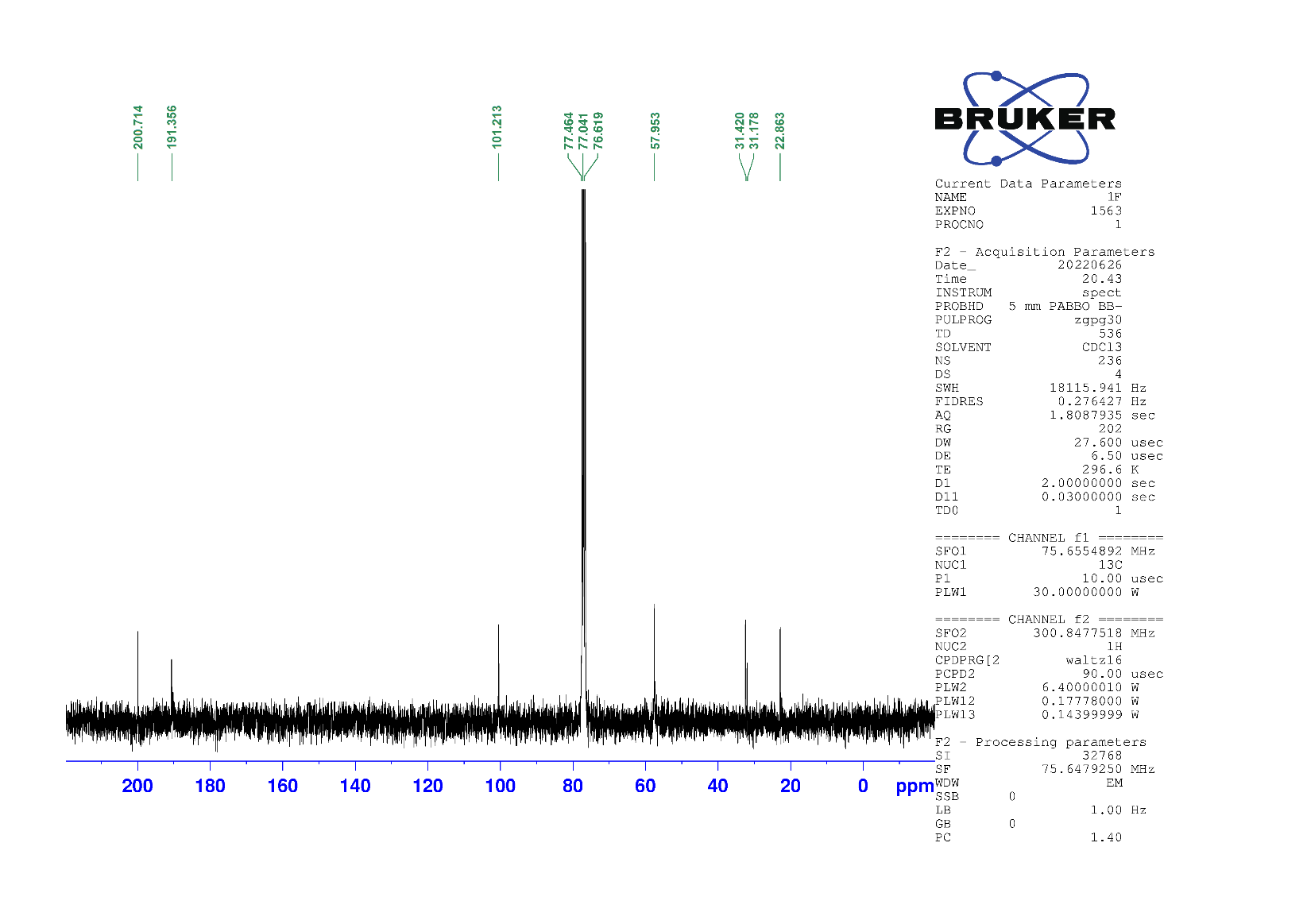
**
